# Early Correlated Network Activity in the Hippocampus: Its Putative Role in Shaping Neuronal Circuits

**DOI:** 10.3389/fncel.2017.00255

**Published:** 2017-08-22

**Authors:** Marilena Griguoli, Enrico Cherubini

**Affiliations:** ^1^European Brain Research Institute (EBRI) “Fondazione Rita Levi-Montalcini” Rome, Italy; ^2^Department of Neuroscience, International School for Advanced Studies Trieste, Italy

**Keywords:** GDPs, network-driven events, postnatal development, hippocampus, GABAergic interneurons, SPWs, depolarizing GABA, chloride transporters

## Abstract

Synchronized neuronal activity occurring at different developmental stages in various brain structures represents a hallmark of developmental circuits. This activity, which differs in its specific patterns among animal species may play a crucial role in *de novo* formation and in shaping neuronal networks. In the rodent hippocampus *in vitro*, the so-called giant depolarizing potentials (GDPs) constitute a primordial form of neuronal synchrony preceding more organized forms of activity such as oscillations in the theta and gamma frequency range. GDPs are generated at the network level by the interaction of the neurotransmitters glutamate and GABA which, immediately after birth, exert both a depolarizing and excitatory action on their targets. GDPs are triggered by GABAergic interneurons, which in virtue of their extensive axonal branching operate as functional hubs to synchronize large ensembles of cells. Intrinsic bursting activity, driven by a persistent sodium conductance and facilitated by the low expression of Kv7.2 and Kv7.3 channel subunits, responsible for *I*_M_, exerts a permissive role in GDP generation. Here, we discuss how GDPs are generated in a probabilistic way when neuronal excitability within a local circuit reaches a certain threshold and how GDP-associated calcium transients act as coincident detectors for enhancing synaptic strength at emerging GABAergic and glutamatergic synapses. We discuss the possible *in vivo* correlate of this activity. Finally, we debate recent data showing how, in several animal models of neuropsychiatric disorders including autism, a GDPs dysfunction is associated to morphological alterations of neuronal circuits and behavioral deficits reminiscent of those observed in patients.

## Introduction

During brain development, neuronal circuits established by a fixed genetic program, regulated already *in utero* by maternal factors (see Reid et al., 2017), undergo refinement though adaptive processes involving experience- or activity-dependent mechanisms such as synapse formation and elimination (Ben-Ari, 2001; Spitzer, 2006). In the visual system, for instance, genetically determined visual projections from the retina to the brain and within the brain among visual areas, following the opening of the eyes are tuned by visual experience into an adult pattern of connectivity (Blankenship and Feller, 2010).

As neurons start to develop synaptic connections and functional circuits become to be defined, spontaneous network-driven events, involving large neuronal populations begin to emerge. This activity differs among animal species. In rodents, it occurs at different developmental stages in various brain structures, including the retina (Galli and Maffei, 1988; Meister et al., 1991), the spinal cord (Landmesser and O’Donovan, 1984), the cerebellum (Watt et al., 2009), the cochlea (Tritsch et al., 2007), the hippocampus (Ben-Ari et al., 1989) and the neocortex (Garaschuk et al., 2000).

In the *in vitro* rodent hippocampus, early synchronized events take the form of giant depolarizing potentials (GDPs; Ben-Ari et al., 1989). GDPs are generated by the interplay between the neurotransmitters GABA and glutamate that, early in postnatal life, are both depolarizing and excitatory (Ben-Ari et al., 1989). They occur at the frequency of 0.05–0.5 Hz and are characterized by large membrane depolarization, lasting several hundreds of milliseconds, with superimposed bursts of action potentials, followed by silent periods. Depolarizing responses are usually subthreshold for action potential generation. They require the activation of a persistent sodium conductance to bring the cell to fire (Sipilä et al., 2006a; Valeeva et al., 2010).

The sustained membrane depolarization activates voltage-dependent calcium channels and N-methyl-D-aspartate (NMDA) receptors with consequent rise of intracellular calcium. This in turn stimulates downstream cascades essential for several developmental functions (Cherubini et al., 1991).

In rats and mice, GDPs disappear towards the end of the first postnatal week, when GABA shifts from the depolarizing to the hyperpolarizing direction. Therefore, GDPs are limited to a transient period and precede more synchronized forms of activity, such as gamma rhythms, known to be involved in high cognitive functions (Buzsáki and Draguhn, 2004). The emergence of gamma oscillations may be favored by the late switch in GABA polarity at axon initial segments of principal cells, as demonstrated in the somatosensory (Khirug et al., 2008) and prefrontal cortex (Rinetti-Vargas et al., 2017).

The depolarizing or hyperpolarizing action of GABA, depends on the intracellular concentration of chloride [Cl^−^]_i_ which is regulated via the cation-chloride importer and exporter NKCC1 and KCC2, respectively. The enhanced membrane expression of KCC2 towards the end of the first postnatal week is responsible for the shift of GABA from the depolarizing to the hyperpolarizing direction (Rivera et al., 1999). Two different splice variants of KCC2 exist: KCC2a and KCC2b. While the expression of KCC2a remains relatively low throughout life, KCC2b is strongly upregulated during postnatal life, particularly in most rostral regions of the CNS, in in both brain region- and species-specific ways. This explains why, immediately after birth, GABA promotes fast hyperpolarizing responses in the spinal cord but not in the hippocampus or in the neocortex (reviewed by Kaila et al., 2014). The developmentally regulated expression of KCC2 is controlled by several factors including membrane trafficking and phosphorylation processes (Kahle et al., 2013; Kaila et al., 2014). Interestingly, KCC2 is also involved in dendritic spines formation independently of its chloride transport function (Li et al., 2007).

The early depolarizing action of GABA is critical for the proper development of cortical neurons. Thus, the premature expression of KCC2 (Cancedda et al., 2007) or the suppression of the excitatory GABAergic input from the zona incerta to cortical pyramidal neurons in the somatosensory and motor cortex (Chen and Kriegstein, 2015), causes a severe impairment of dendritic arborization. It is worth noting that the balance between NKCC1 and KCC2 is highly labile and it may return to an immature state after seizures, spinal cord lesions, and other pathological conditions (Ben-Ari et al., 2012; Kaila et al., 2014).

The aim of this review article is to provide the background for the functional role of GABAergic signaling and particularly of spontaneously occurring network-driven synaptic events such as GDPs in brain maturation. We will discuss also how GDPs dysfunctions may lead to severe alterations in synaptic wiring and neurodevelopmental disorders.

## Mechanisms of GDPs Generation

GDPs are synaptic-driven events: they require the concomitant activation of a relatively small number of cells within a local neuronal circuit. They persist in small tissue islands, isolated from the rest of the hippocampus, containing few hundreds of neurons (Khazipov et al., 1997; Garaschuk et al., 1998; Bolea et al., 1999). Although GDPs can independently initiate from different hippocampal regions, in the CA3 area they are facilitated by the extensive network of recurrent excitatory connections among interneurons and principal cells (Menendez de la Prida et al., 1998). In this area, GDPs are also facilitated by the presence of intrinsic bursts that can drive other neurons to fire (Sipilä et al., 2005; Safiulina et al., 2008). Hence, in the presence of ionotropic synaptic antagonists, spontaneous voltage-dependent bursts of spikes, can be unveiled in CA3 principal cells. Intrinsic bursting activity, which plays a permissive role in generation of network-driven events, is initiated by a slow regenerative depolarization driven by a persistent sodium conductance (Sipilä et al., 2005, 2006a). Intrinsic bursts are also favored by the low expression of Kv7.2 and Kv7.3 channel subunits, responsible for *I*_M_ in the hippocampus. The low density of *I*_M_ in neonatal CA3 pyramidal cells facilitate intrinsic bursts that in comparison with juvenile or adult neurons are more robust, last longer and occur more regularly (Safiulina et al., 2008).

CA3 pyramidal neurons trigger GDPs but are not required for their generation since oscillatory activity can be recorded at lower frequency in the CA1 region, surgically isolated from the CA3 (Ben-Ari et al., 2007). In addition, growing evidence suggests that extrasynaptic transmission contributes to propagating waves of depolarization in developing networks. After being released from presynaptic nerve terminals, neurotransmitters can spill out to activate extrasynaptic receptors located on postsynaptic, presynaptic, neighboring cells and glia. The activation of high affinity extrasynaptic GABA_A_ receptors by ambient GABA generates a tonic GABA_A_-mediated conductance that contributes to depolarize targeted cells to the voltage window where intrinsic bursts are generated (Sipilä et al., 2005, 2009), and to enhance the glutamatergic drive to principal cells (Marchionni et al., 2007). It is worth noting that, in the absence of glycinergic synapses, the newborn hippocampus is endowed of strychnine-sensitive anion-permeable glycine receptors possibly activated by endogenous glycine, taurine and β-alanine (Ito and Cherubini, 1991; Sipilä et al., 2014). The glycine receptor mediated tonic conductance, controlled by glycine transporter 1, exerts an inhibitory action on GDPs despite a depolarizing chloride driving force (Sipilä et al., 2014). Whether also extrasynaptic glutamate receptors activated by ambient glutamate participate to GDPs generation remains to be established.

Interestingly, in a recent study evidence has been provided that GABA action during GDPs is dynamically controlled by the membrane potential in such a way that is excitatory at their onset and inhibitory at their peak (Khalilov et al., 2015). This inhibitory effect would prevent generation of seizures in the immature hippocampal network.

The circuit mediating network-driven oscillations comprises principal cells and interneurons and therefore GDPs express both GABAergic and glutamatergic components. The magnitude of the GABAergic conductance however, exceeds that of the glutamatergic one and GDP’s reversal is close to E_GABA_ (Ben-Ari et al., 1989; Bolea et al., 1999). The glutamatergic component can be unveiled by blocking the GABAergic one with an intracellular solution containing fluoride, which poorly permeates GABA_A_ receptor channels. In this condition, the reversal of GDPs is close to zero, the equilibrium potential for AMPA receptors (Bolea et al., 1999). Local GABAergic interneurons usually drive principal cells to fire, as demonstrated by the temporal relationship between glutamatergic and GABAergic inputs. The first evidence that GABA released from GABAergic interneurons drives principal cells was provided by Mohajerani and Cherubini (2005) who, using organotypic hippocampal slice cultures, demonstrated, by holding two neighboring CA3 principal cells at the reversal potentials for glutamate (~0 mV) and GABA (~−70 mV), respectively, that the GABAergic component of GDPs always precedes the glutamatergic one by several milliseconds. Later on, using network dynamics imaging, online reconstruction of functional connectivity and targeted whole-cell recordings from immature hippocampal slices, Bonifazi et al. (2009) demonstrated that GABAergic interneurons with large axonal branching, operate as functional hubs to synchronize large ensembles of cells (Bonifazi et al., 2009).

**Figure 1:**
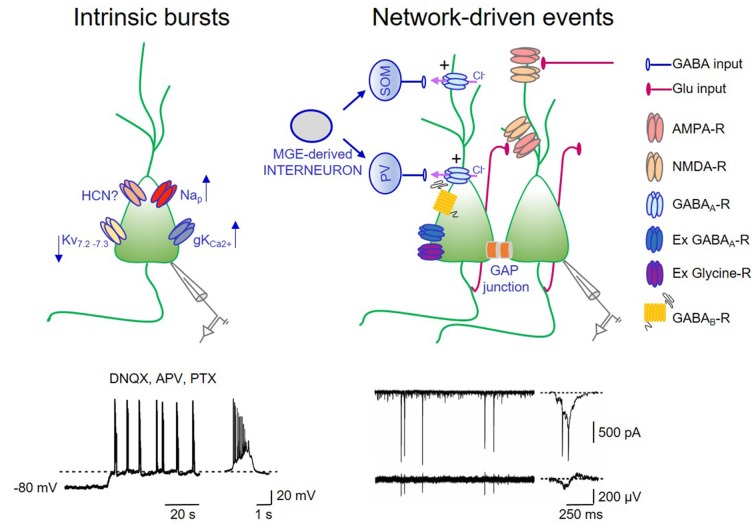
Intrinsic bursts exert a permissive role in giant depolarizing potentials (GDPs) generation. On the left: schematic drawing of a pyramidal cell endowed with some of the ionic channels contributing to up- and down regulate intrinsic bursts. Na_p_: voltage-dependent sodium channel mediating persistent sodium current (I-Nap); Kv7.2–7.3: potassium channel responsible for *I*_M_; hyperpolarization-activated cyclic nucleotide (HCN)-gated channel mediating *I*_h_; gKCa^2+^: calcium dependent potassium channel mediating the slow after hyperpolarization. In the trace below: whole cell patch clamp recording (in current clamp configuration) from a P3 CA3 principal cell, in the presence of DNQX, APV, PTX to block synaptic transmission. A membrane depolarization from −80 mV to the voltage window where Na_p_ channels are activated (dashed line) triggers bursting activity (a single burst shown on the right in an expanded time scale). Intrinsic bursts are facilitated by the low expression of *I*_M_. The pacemaking role of *I*_h_ in bursting activity is unclear (see text). Calcium rise during repeated action potentials within the burst open calcium-dependent potassium channels responsible for bursts termination. On the right: schematic drawing of the local hippocampal circuit responsible for GDPs. Medial ganglionic eminence (MGE) derived interneurons (gray) give rise to somatostatin (SOM) and parvalbumin (PV)-positive interneurons (blue) innervating the distal and proximal dendrites of pyramidal cells, respectively. CA3 principal cells (green) are connected via recurrent collaterals (red) and through gap junctions. They receive also glutamatergic inputs from entorhinal cortex (EC) and from the controlateral hippocampus (red). At this developmental stage, the majority of GABAergic synaptic contacts on principal cells and interneurons are depolarizing and excitatory (+) because of the outwardly directed flux of chloride ions. Gap junctions favor network synchronization and GDPs occurrence. In addition, GDPs are modulated by extrasynaptic GABA_A_ and glycine receptors. Activation of GABA_B_ receptors by massive release of GABA during GDPs, together with the activation of calcium-dependent potassium channels are responsible for GDPs termination. Below: whole cell patch clamp recording (in voltage clamp configuration) of network-driven events (GDPs) from a P5 CA3 principal cell (upper trace) and associated local field potentials (bottom trace). These events are shown on the right in an expanded time scale.

Using “genetic fate mapping” to selectively label GABAergic neurons on the basis of their place and time of origin, Picardo et al. (2011) found that, a subpopulation of superconnected hub neurons, characterized by an exceptionally widespread axonal arborization is generated earlier than other interneurons. These cells may persist in adulthood as putative long-range GABAergic projecting cells (Jinno et al., 2007). In agreement with the dendritic localization of immature GABAergic synapses (Gozlan and Ben-Ari, 2003), early generated interneurons target mainly dendrites. These interneurons usually contain somatostatin. However, this population is highly heterogeneous and comprises also parvalbumin-positive perisomatic basket cells. Recently, optogenetic tools have been used to address the question of which interneurons, among those generated during embryonic development from the caudal or medial ganglionic eminence (CGE or MGE), respectively, play a dominant role in GDPs generation in the neonatal mouse hippocampus *in vitro* (Wester and McBain, 2016). Light activation of MGE-derived interneurons with archaerhodopsin suppresses GDPs in a region-specific manner, whereas activation of CGE-derived interneurons has a small impact on GDPs. Interestingly, as early generated neurons (Picardo et al., 2011), MGE-derived interneurons have a higher rate of synaptic connectivity and give rise to interneurons containing mainly somatostatin and parvalbumin (Figure 1).

As for population bursts generated in the CA3 region of the adult hippocampus by suppression of inhibition, GDPs occur in a stochastic manner when neuronal excitability attains, within a restricted period of time, a certain threshold (de la Prida et al., 2006). Simultaneous recordings from pairs of CA3 pyramidal neurons have shown that GDPs are preceded by an increased frequency of spontaneously occurring synaptic events able to drive a sufficient number of cells to fire (Menendez de la Prida and Sanchez-Andres, 1999). In the case of GDPs, synaptic interactions are facilitated by the excitatory action of GABA. Using knock-in mice with conditional expression of channelrhodopsin-2 in GABAergic interneurons, Valeeva et al. (2016) have clearly demonstrated that, activation of interneurons by light, evokes in P2–P8 hippocampal slices an increase in frequency of glutamatergic excitatory postsynaptic currents (EPSCs). In line with the excitatory-to-inhibitory switch of GABA action (Ben-Ari et al., 1989), light caused an increase in EPSCs frequency at P2–P8 and a decrease at P9–P15.

Additional factors that may contribute to trigger GDPs in the immature hippocampus are: (i) the low expression of Kv7.2 and Kv7.3 channels, responsible for the non-inactivating, low-threshold M current (*I*_M_) that in adulthood controls spike after-depolarization and burst generation (Yue and Yaari, 2004); (ii) the slow activating inwardly rectifying cationic current *I*_h_ mediated by HCN channels highly expressed in the hippocampus from birth, known to facilitate network oscillations (Pape, 1996). In a previous study, we found that a low concentration (0.3 mM) of the *I*_h_ blocker cesium was able to block GDPs in hilar interneurons (Strata et al., 1997) suggesting a pacemaking role of *I*_h_ in GDPs. In contrast, Sipilä et al. (2006a), using a higher concentration of cesium (0.5–2 mM), observed an increase in frequency of GDPs, probably caused by the inhibition of selective potassium channels. Whether this discrepancy depends on the different concentrations of cesium or other factors is presently unknown. As in Strata et al. (1997), GDPs were reduced by ZD 7288, another *I*_h_ antagonist (Bender et al., 2005). However, at concentrations used (1–50 μM), this compound may have unspecific effects (Chevaleyre and Castillo, 2002). The observation that mice lacking HCN channels can still generate GDPs (Bender et al., 2005), strongly suggest that *I*_h_ is not critically involved in these events; and (iii) gap junctions. They constitute a way of signaling particularly well developed in immature neurons. In the hippocampus, neuronal coupling via gap junctions, contributes to trigger GDPs as demonstrated by their disruption upon gap junctions uncoupling with octanol (Strata et al., 1997; Figure 1). However, these results should be taken with caution in view of the fact that octanol has several non-specific effects, including blockade of voltage-gated calcium channels and transmitter release (Tovar et al., 2009).

Interestingly, chemokine stromal cell-derived factor-1-alpha (SDF-1 or CXCL12), the natural ligand for chemokine motif receptor 4 (CXCR4), known to play an important role in brain development, decreases GDPs frequency, an effect prevented by T140, a CXCR4 receptor antagonist, suggesting that SDF-1 alpha modulates GDPs via CXCR4. The inhibitory action of SDF-1 alpha on GDPs may reflect a potential mechanism for chemokine regulation of neural development early in postnatal life (Kasyanov et al., 2006).

How do GDPs terminate? Early studies from CA3 pyramidal cells, have suggested that burst firing of action potentials at the top of GDPs, induces a transient elevation of intracellular calcium, which activates a calcium-dependent potassium conductance responsible for the slow after-hyperpolarization (AHP) that follows GDPs (Ben-Ari et al., 1989; Sipilä et al., 2006a). The duration of the AHP would depend on the number of action potentials and the amount of intracellular calcium. However, other evidences indicate that activation of postsynaptic GABA_B_ receptors by massive release of GABA during GDPs may contribute to the AHP (McLean et al., 1996; de la Prida et al., 2006; Fiorentino et al., 2009). Immediately after birth, postsynaptically expressed GABA_B_ receptors are absent and monosynaptically evoked GABAergic responses in CA3 pyramidal cells and neocortical neurons lack the late GABA_B_-mediated component. This starts appearing as early as at the postnatal day P3 (Luhmann and Prince, 1991; Fukuda et al., 1993; Gaiarsa et al., 1995; Caillard et al., 1998; Nurse and Lacaille, 1999; Verheugen et al., 1999). The possibility therefore that, during the first postnatal days, postsynaptic GABA_B_ receptors are activated only by massive release of GABA during GDPs cannot be excluded. In favor of this hypothesis is the observation that blockade of GABA_B_ receptors prolong GDPs and transform them in interictal- and ictal-like discharges (McLean et al., 1996). A recent study (Khalilov et al., 2017), has confirmed that the AHP following GDPs involves a calcium-dependent potassium conductance activated by calcium rise during burst firing and a GABA_B_ receptor-mediated potassium conductance activated by the release of GABA. The cooperation of these two complementary inhibitory postsynaptic mechanisms may contribute to terminate GDPs.

## Correlated Network Activity Is Crucial for Enhancing Synaptic Efficacy at Immature Gabaergic and Glutamatergic Connections

How neuronal connectivity emerges early in postnatal life constitutes a fundamental question in developmental neurobiology. After their proliferation, neurons migrate to the proper position and differentiate through processes involving several cell-intrinsic and extrinsic signals including the neurotransmitter GABA, which, at this developmental stage, works mainly as a trophic factor (Wang and Kriegstein, 2009). Interestingly, GABA_A_ receptors are present on neuronal progenitors before synapse formation. Extrasynaptic GABA_A_ receptors behave like sensors for “ambient” GABA, released in a calcium and SNARE-independent way by growth cones and astrocytes. This neurotransmitter enables to activate, in a paracrine fashion, cells located at substantial distances from the releasing sites (Demarque et al., 2002). Synapses develop later and, at least in principal cells of the hippocampus, GABAergic connections occur before glutamatergic ones. Their development strictly correlates with the level of dendritic arborization (Tyzio et al., 1999; Khazipov et al., 2001; Ben-Ari et al., 2007). At later developmental stages, spontaneously active GABAergic and glutamatergic synapses give rise to GDPs, which, by temporally linking neuronal ensembles, facilitate synaptic plasticity.

In previous studies, the hypothesis was tested that GDPs-associated calcium transients act as coincident detectors to persistently enhance synaptic efficacy at emerging GABAergic and glutamatergic connections (Kasyanov et al., 2004; Mohajerani et al., 2007). To this aim, a pairing procedure was developed, consisting in stimulating for a short period of time (5 min) mossy fibers (MF), the axons of granule cells, with the rising phase of spontaneously occurring GDPs, in such a way that the two events (GDPs and MF inputs) occurred simultaneously. Immediately after birth, MF releases mainly GABA (Safiulina et al., 2006, 2010). Pairing GDPs with MF stimulation caused a persistent increase in amplitude of MF-evoked synaptic currents that was associated to a reduced incidence of failures of transmitter release. This form of long-term potentiation (LTP) was maintained through a presynaptic increase in the probability of GABA release as indicated by the decrease in paired pulse ratio. In fact, an increase in transmitter release in response to the first stimulus leads to a decreased amount of transmitter released in response to the second stimulus. In the absence of pairing, these changes do not occur. This form of LTP has a clear temporal and spatial specificity. First, LTP declined to baseline level when a delay of few seconds intervened between GDPs and synaptic stimulation. Second, GDPs-induced synaptic potentiation was generally restricted to the paired input and only occasional spread to the unpaired one. The mechanism of pairing-induced LTP involved elevation of intracellular calcium in the postsynaptic cell through voltage-gated calcium channels, since it was prevented by intracellular BAPTA and by bath application of the calcium channel blocker nifedipine. Although, LTP seems to rely mainly in a presynaptic increase in GABA release from MF terminals, as indicated by pairing-induced changes in the paired-pulse ratio, postsynaptic modifications such as insertion of new receptors in the subsynaptic membrane cannot be excluded. Both processes may contribute to the appearance of synaptic responses at apparently silent connections. The immature brain is known to express an elevated number of silent synapses. These are synapses that do not conduct at rest either because the probability of neurotransmitter released is too low to activate low affinity receptors or because they are unable to detect the release of neurotransmitters due to the lack of receptors on the subsynaptic membrane (Durand et al., 1996; Gasparini et al., 2000). Conversion of silent synapses into conductive ones represents the most common mechanism of LTP (Voronin and Cherubini, 2004). In line with Kasyanov et al. (2004), previous data from the immature hippocampus have shown that repeated bursts of action potentials, applied at low frequency to CA3 principal cells, are able to potentiate GABA_A_-mediated synaptic currents in an NMDA-independent way (Caillard et al., 1999; Gubellini et al., 2001, 2005). However, in these studies the origin of GABAergic input was not identified.

Similarly to MF-CA3 synapses, GDPs act as coincident detectors to enhance synaptic strength at immature glutamatergic CA3-CA1 connections (Mohajerani et al., 2007). Also in this case, pairing GDPs with Schaffer collateral stimulation induced a persistent increase in amplitude of glutamatergic currents. LTP induction was postsynaptic since it required calcium rise in the postsynaptic cell via voltage-dependent calcium channels. However, its expression was presynaptic as suggested by pairing-induced decrease in failure rate, in paired pulse facilitation, and increase in the inversed square of the coefficient of variation, all indices of presynaptic change in release probability. This implies a cross-talk between the post- and presynaptic sites via a retrograde messenger. BDNF, possibly secreted from the postsynaptic neuron during GDPs-induced burst firing, was identified as the retrograde signal. Thus, pairing-induced synaptic potentiation was prevented by scavengers of endogenous BDNF or by tropomyosin-related kinase receptor B (TrkB) antagonists. In addition, exogenously applied BDNF mimicked pairing-induced synaptic potentiation.

## The *In Vivo* Counterpart of GDPs

The predominant rhythm observed at P3–P6 in the CA1 region of the hippocampus of both anesthetized and awake rats consists in sharp wave (SPW) oscillations occurring at ~0.1 Hz, followed by multiunit bursts lasting from 0.5 s to 3 s (Leinekugel et al., 2002; Buzsáki, 2015). Starting from the second postnatal week, bursts associated to SPWs disappear while faster oscillations including dentate spikes, theta, gamma and ripples emerge (Lahtinen et al., 2002; Leinekugel et al., 2002; Buhl and Buzsáki, 2005).

**Figure 2:**
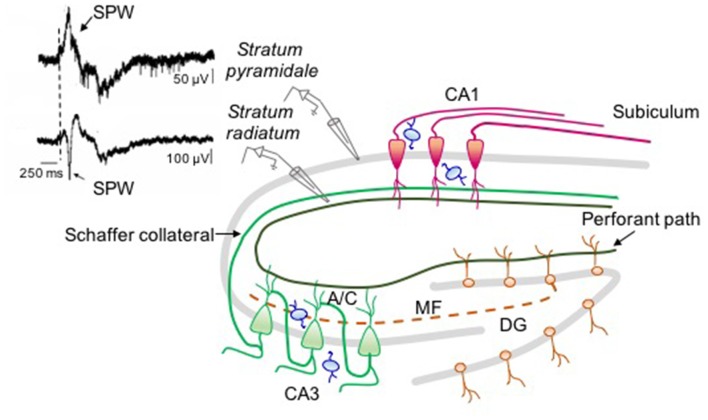
Neuronal circuit involved in sharp wave (SPW) generation in the intact hippocampus. On the right: schematic drawing representing the circuit responsible for SPW generation. On the left: simultaneous extracellular field recordings obtained *in vivo* (at P6) from *stratum pyramidale* (upper trace) and *stratum radiatum* (bottom trace) of CA1 hippocampal area (modified from Leinekugel et al., 2002). In *stratum pyramidale* the SPW (arrow) is followed by a burst of firing. The largest amplitude of the negative deflection (note the different calibration of the two traces) occurs in the middle of *stratum radiatum* where CA3 principal cells (green) make synaptic contacts with CA1 neurons (red) through Schaffer collateral and associative commissural (A/C) inputs suggesting, as GDPs, a CA3 origin. The contribution of immature mossy fiber path (dashed line) and perforant path from EC cannot be excluded. All glutamatergic inputs are regulated by local interneurons (blue).

The level of synchronization and the frequency of SPWs are reminiscent of GDPs observed in hippocampal slices (Ben-Ari et al., 1989). Hippocampal bursts occur mainly during immobility periods, sleep, and feeding. These are often associated to twitches of skeletal muscles, indicating, as in the cortex, the involvement of a sensory feedback mechanism (Khazipov and Luhmann, 2006). During crawling, an irregular firing replaces bursting activity in the absence of background oscillations (Mohns and Blumberg, 2008). Like GDPs, SPWs bear double glutamatergic and GABAergic synaptic components (Leinekugel et al., 2002). The glutamatergic component probably reflects the excitatory drive from the entorhinal cortex (EC) and from the Schaffer collateral of CA3 principal cells, already present and functional at P2 (Supèr and Soriano, 1994; Figure 2). EC inputs may carry sensory feedbacks from neocortical areas (Mohns and Blumberg, 2010) while CA3 may carry information from the intrinsic hippocampal assemblies. Synaptic input from the CA3 area may prevail since, the amplitude/depth profile of SPWs reaches a peak in *stratum radiatum* where CA3 axon terminals make synaptic contacts with CA1 dendritic spines (Leinekugel et al., 2002). Moreover, synaptic responses evoked by stimulating the ventral hippocampal commissure show the same amplitude/depth distribution of SPWs, confirming the involvement of intra-hippocampal inputs (Leinekugel et al., 2002; Figure 2). Although, the glutamatergic component prevails, the GABAergic one seems to play a key role in promoting SPWs as the intraperitoneal administration of bumetanide completely abolishes them (Sipilä et al., 2006b). Bumetanide may act via a peripheral action since apparently with low doses applied i.p., it does not have effect *in vivo* on hippocampal and cortical neurons (Puskarjov et al., 2014).

While a large body of evidence suggests that early in postnatal life GABA exerts a depolarizing and excitatory effect on its targets *in vitro*, its action *in vivo* is still a matter of debate. Thus, combining electrophysiological and imaging techniques, Kirmse et al. (2015) have shown that in anesthetized animals, exogenously applied GABA from a pressure pipette controls network activity through shunting inhibition in spite its depolarizing effects in the majority of immature neurons. In this case, GABA-induced membrane depolarization would not attain the threshold for action potential generation. Similarly, using an optogenetic approach, Valeeva et al. (2016) have demonstrated that photo-stimulation of GABAergic interneurons expressing channelrodopsin (at P3–P9), produces a reduction in frequency of spontaneous glutamatergic events, suggesting an inhibitory effect. Photorelease of GABA from GABAergic interneurons would inhibit the firing of principal cells leading to a depression of spontaneously occurring EPSCs. In contrast to this view, a recent *in vivo* study has clearly demonstrated that, the depolarizing action of GABA induces via the activation of T-type voltage dependent calcium channel, calcium rise, leading to an increased number of gephyrin puncta and dendritic spines in layer 2/3 neocortical pyramidal neurons. This suggests that the depolarizing and excitatory action of GABA is involved in “the novo” synaptogenesis (Oh et al., 2016). Although the reasons for these discrepancies are still unknown, the possibility that different experimental conditions including the effects of anesthetics, electrolyte composition of the extracellular medium, temperature, gas exchanges may modify passive and active membrane properties (i.e., resting membrane potential and action potential threshold) of *in vivo* recorded neurons cannot be excluded. However, indirect evidence that GABA may exert a depolarizing and excitatory action also *in vivo* is provided by the experiments in which inhibiting the chloride importer NKCC1 with systemic administration of bumetanide suppress SPWs (Sipilä et al., 2006b). The observation that the intraperitoneal delivery of bumetanide is able to prolong critical-period plasticity in visual cortical circuits without affecting the overall development of the visual system, an effect that involves BDNF and extracellular matrix perineuronal nets, further supports this view (Deidda et al., 2015). In favor of the depolarizing and excitatory action of GABA *in vivo* is also the paradoxical excitatory effect exerted by benzodiazepines in neonates affected by autism spectrum disorders (ASDs) and epilepsy (Bruining et al., 2015).

Although difficult to compare, early synchronized activity recorded *in vivo* from the rat hippocampus during the first postnatal week (Leinekugel et al., 2002) is reminiscent of that observed in the electroencephalogram (EEG) of premature babies. It is worth noting that mice and rats born at an early stage of brain development corresponding to the second half of human gestation (Clancy et al., 2001). The EEG of 20 weeks post-conception babies has been defined by Dreyfus-Brisac et al. (1956) “*tracé discontinue*” because of the alternation between activity’s bursts synchronized across the two hemispheres and long silent periods (lasting for minutes). During maturation, silent periods between bursts decrease and, starting from the 30th week post-conception, the “*tracé discontinue*” evolves into the “*tracé alternant*” (Stockard-Pope et al., 1992).

## GDPs Dysfunction in Animal Models of Neurodevelopmental Disorders

Early changes in GABA action (from depolarizing and excitatory to hyperpolarizing and inhibitory) may impair GDPs expression and circuit formation. These alterations are often present in animal models of neurodevelopmental disorders including ASDs (Tyzio et al., 2014). Interestingly, in a small number of cases, ASDs have been found to be associated with single mutations in genes involved in synapse function. Therefore, these forms of ASDs can be considered synaptopathies (Südhof, 2008). Some of these involve adhesion molecules of the neuroligin (NL)/neurexin (NRX) families, which ensure the cross talk between the post and presynaptic specializations. Neonatal mice carrying the R451C mutation of NL3 (NL3^R451C^ knock-in mice), found in a family with two autistic children (Jamain et al., 2003), exhibit behavioral deficits reminiscent of those present in autistic patients. These mice show an increase in GDP frequency probably dependent on the enhanced excitatory GABAergic drive to principal cells (Pizzarelli and Cherubini, 2013). In another mouse model of idiopathic autism (BTBR T+tf/J), whose genetic background is still under investigation (Jones-Davis et al., 2013), the reduced neuronal excitability within the CA3 hippocampal circuit, leads to a reduced frequency of GDPs and a persistent deficit in behavioral functions (Cellot et al., 2016).

A reduced frequency of GDPs was detected also in CA3 principal cells in hippocampal slices from neonatal mice carrying the human mutation (R43Q) of the γ2 subunit of GABA_A_ receptors, known to have long-lasting effects on seizures susceptibility during a critical developmental period (Chiu et al., 2008). As compared to controls, mice heterozygous for this mutation show a significant decrease in GDPs frequency associated to a reduction in amplitude and frequency of spontaneous GABAergic and glutamatergic postsynaptic currents (Vargas et al., 2013). Interestingly systemic administration of bumetanide to control animals mimicked the effects of the γ2^R43Q^ epilepsy mutation and lowered the threshold for thermal seizures (Hill et al., 2011) as in patients affected by febrile convulsions (Wallace et al., 2001). These data suggest that a reduced expression of GDPs in the early neonatal period leads to structural impairment of neuronal networks.

Furthermore, GDPs are sensitive to psychoactive agents as alcohol, particularly deleterious for babies, if assumed during pregnancy (Riley et al., 2011). For instance, ethanol (EtOH) exposure potently excites immature neuronal networks by increasing GDP frequency in the CA3 region of the neonatal hippocampus (Galindo et al., 2005). GDPs do not develop tolerance to the modulatory effect of EtOH since the effect does not change with the duration of EtOH’ exposure (Galindo and Valenzuela, 2006). In rats, the intraperitoneal injection of EtOH during the first post-natal week induces weaker synchronization of neuronal activity during GDP, effect that is not reversible with time (Zakharov et al., 2016).

It is important to stress that a causal link between GDPs disruption and behavioral deficits observed in animal models on neuropsychiatric disorders has not been proven yet and the possibility that alterations of GDPs expression are the consequence and not the cause of the above mentioned disorders cannot be excluded. Obviously, these are not mutually exclusive alternatives.

## Conclusions

Although in the last years significant progresses have been made to understand the mechanisms of GDPs generation and their functional role in modifying synaptic strength at emerging GABAergic and glutamatergic connections (Ben-Ari et al., 2012), their role in the wiring of neuronal circuits is still unknown. Many questions are still open. For instance, are pairing-induced changes in synaptic efficacy associated to structural modifications including changes in dendritic morphology, number of branching, spine types and density? Are SPWs *in vivo* counterpart of GDPs? Which circuits are involved? Combined optogenetic tools and *in vivo* recordings from the hippocampus and neocortex of freely moving animals may contribute to unveil the spatial and temporal contribution of selective glutamatergic and GABAergic pathways to SPW/GDP generation. In addition, optical imaging techniques may help elucidating, at mesoscale levels, brain activity *in vivo* (McVea et al., 2016). This would allow estimating the temporal dynamics of oscillations and their propagation in the immature hippocampus and neocortex.

## Author Contributions

MG and EC wrote the article and MG generated the Figures.

## Conflict of Interest Statement

The authors declare that the research was conducted in the absence of any commercial or financial relationships that could be construed as a potential conflict of interest.
